# Supplementary figures and images for: Identification and Characterization of an OSH1 Thiol Reductase from Populus Trichocarpa
Source: Cells. 2019 Dec 27;9(1):76. doi: 10.3390/cells9010076 (PMC7017176; doi:10.3390/cells9010076)

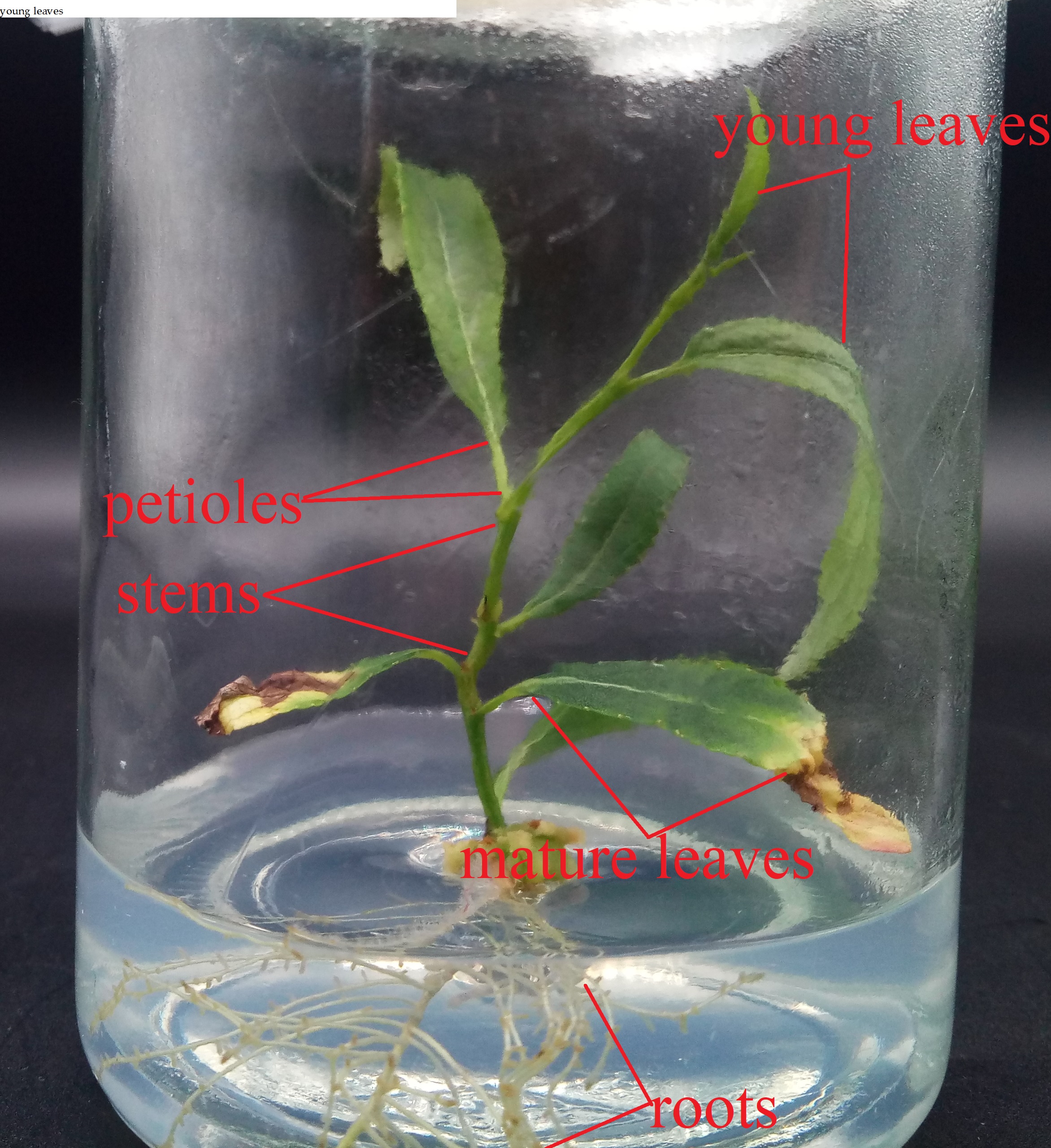

Supplement: Supplementary file 1 [file cells-09-00076-s001.zip › Supplementary/Supplemental Figure 1.jpg]

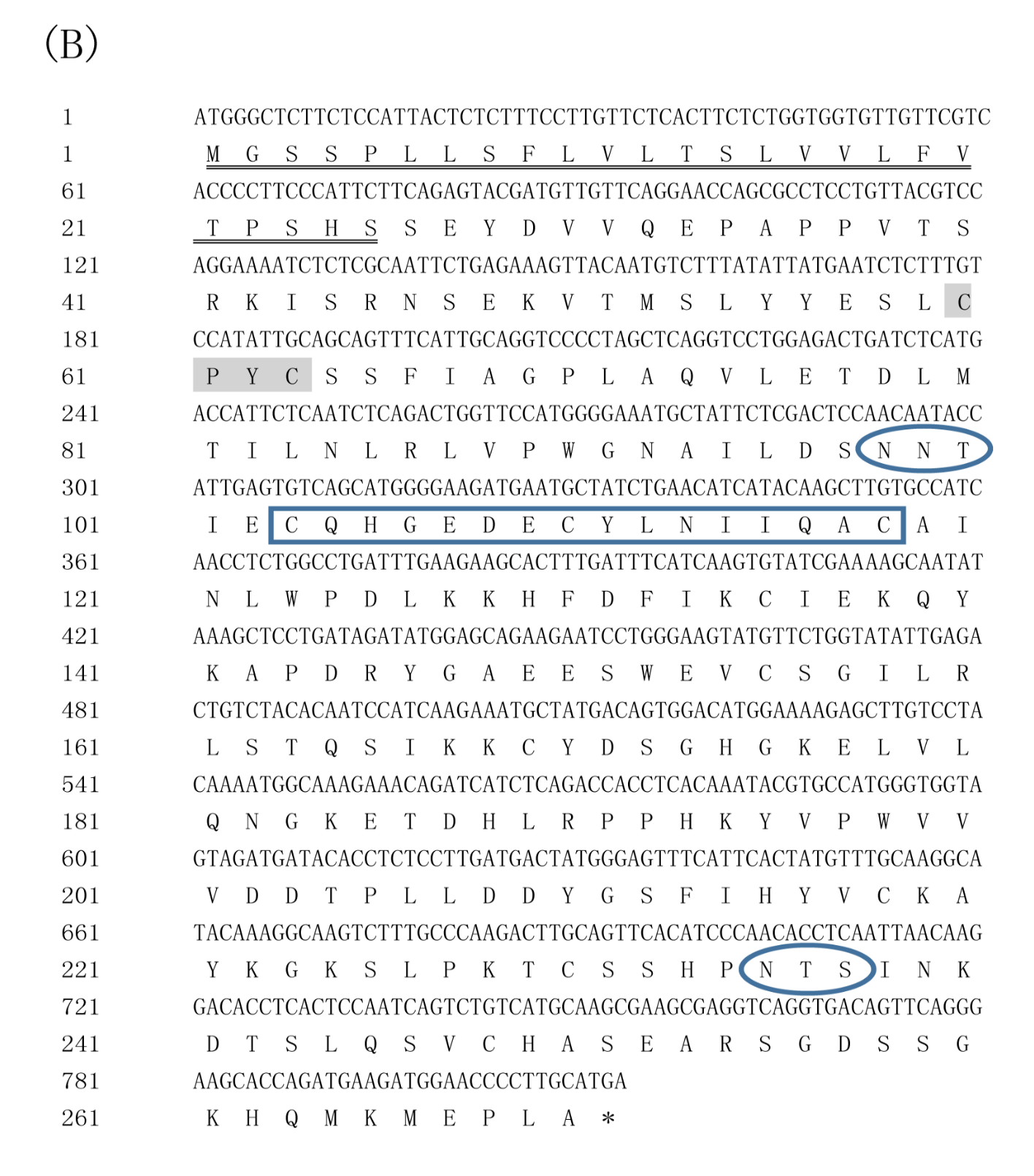

Supplement: Supplementary file 1 [file cells-09-00076-s001.zip › Supplementary/Supplemental Figure 2.tif]

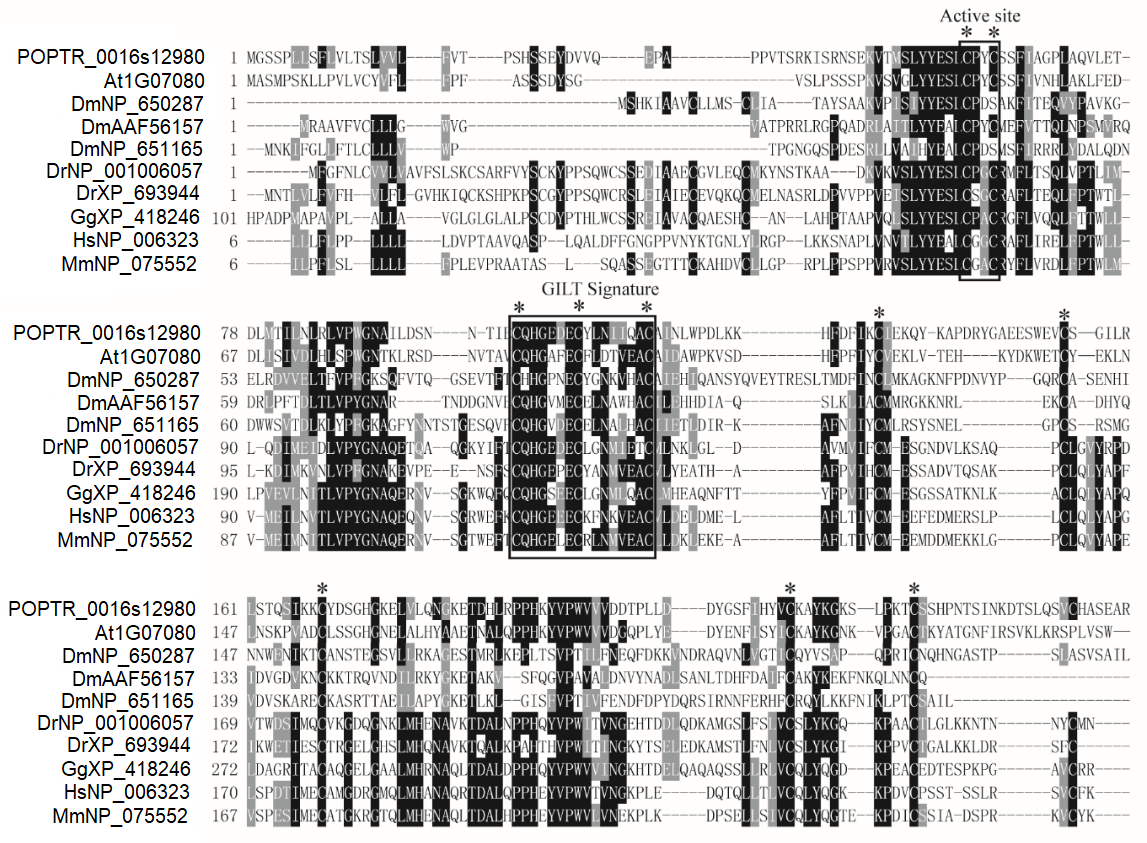

Supplement: Supplementary file 1 [file cells-09-00076-s001.zip › Supplementary/Supplemental Figure 3.tif]

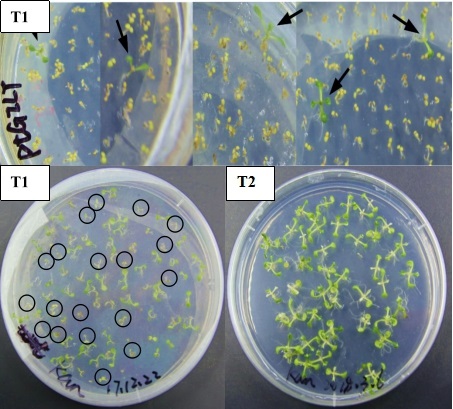

Supplement: Supplementary file 1 [file cells-09-00076-s001.zip › Supplementary/Supplemental Figure 4.jpg]

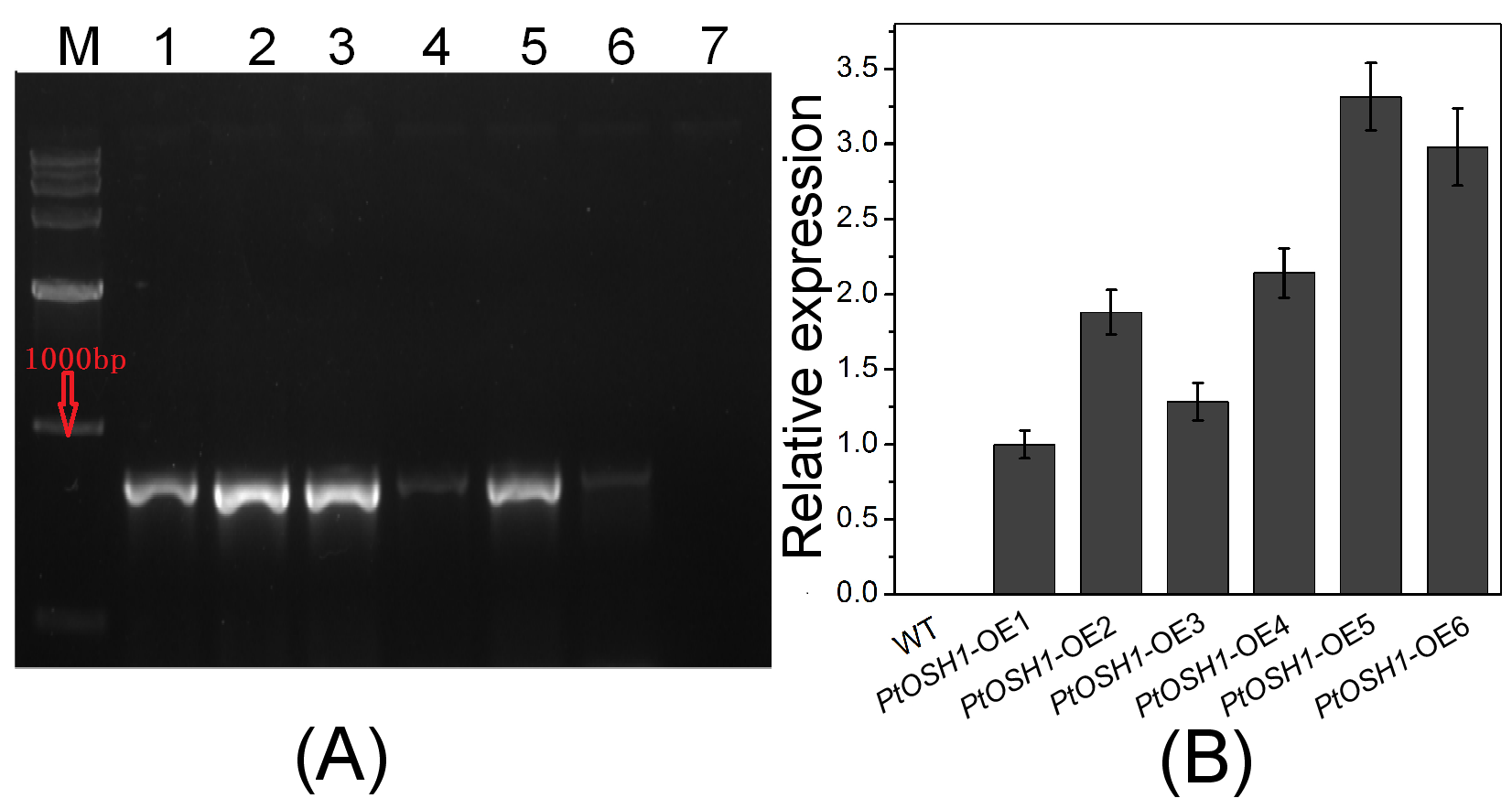

Supplement: Supplementary file 1 [file cells-09-00076-s001.zip › Supplementary/Supplemental Figure 5.tif]

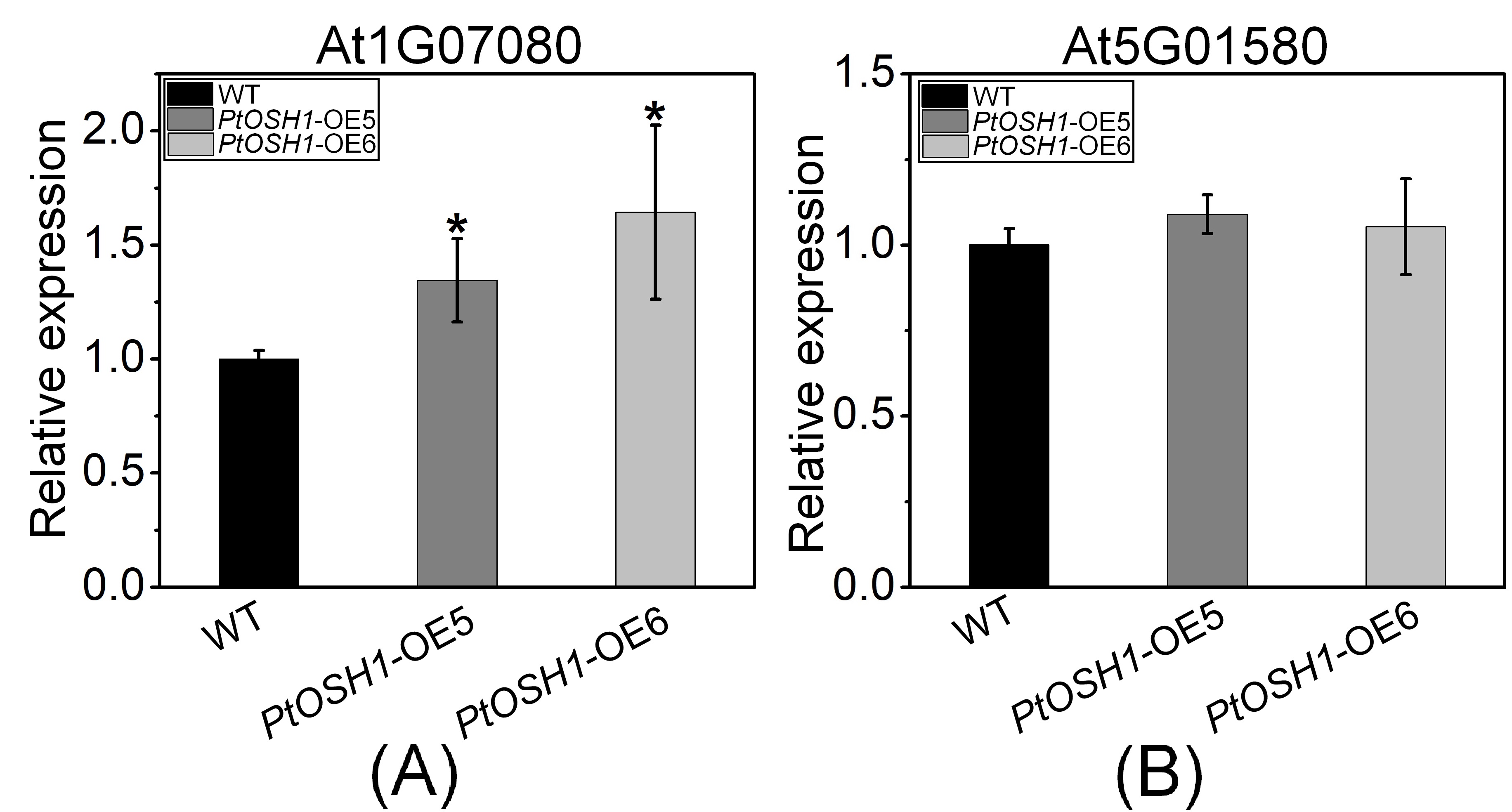

Supplement: Supplementary file 1 [file cells-09-00076-s001.zip › Supplementary/Supplemental Figure 6.jpg]
